# Inhibition of U4 snRNA in Human Cells Causes the Stable Retention of Polyadenylated Pre-mRNA in the Nucleus

**DOI:** 10.1371/journal.pone.0096174

**Published:** 2014-05-05

**Authors:** Anne Hett, Steven West

**Affiliations:** The Wellcome Trust Centre for Cell Biology, University of Edinburgh, Edinburgh, United Kingdom; Rutgers New Jersey Medical School, United States of America

## Abstract

Most human pre-mRNAs contain introns that are removed by splicing. Such a complex process needs strict control and regulation in order to prevent the expression of aberrant or unprocessed transcripts. To analyse the fate of pre-mRNAs that cannot be spliced, we inhibited splicing using an anti-sense morpholino (AMO) against U4 snRNA. As a consequence, splicing of several selected transcripts was strongly inhibited. This was accompanied by the formation of enlarged nuclear speckles containing polyadenylated RNA, splicing factors and the nuclear poly(A) binding protein. Consistently, more polyadenylated pre-mRNA could be isolated from nucleoplasmic as well as chromatin-associated RNA fractions following U4 inhibition. Further analysis demonstrated that accumulated pre-mRNAs were stable in the nucleus and that nuclear RNA degradation factors did not re-localise to nuclear speckles following splicing inhibition. The accumulation of pre-mRNA and the formation of enlarged speckles were sensitive to depletion of the 3′ end processing factor, CPSF73, suggesting a requirement for poly(A) site processing in this mechanism. Finally, we provide evidence that the pre-mRNAs produced following U4 snRNA inhibition remain competent for splicing, perhaps providing a biological explanation for their stability. These data further characterise processes ensuring the nuclear retention of pre-mRNA that cannot be spliced and suggest that, in some cases, unspliced transcripts can complete splicing sometime after their initial synthesis.

## Introduction

Most human pre-mRNAs contain multiple introns that are removed by splicing. The splicing process involves five small nuclear (sn) RNAs and well over a hundred associated factors [1]. It begins with base pairing between U1 snRNA and the 5′ splice site. Subsequently, the 3′ splice site is recognised by U2AF35 and 65 before U2 snRNA base-pairs with the branch-point. U4, U5 and U6 snRNAs are then recruited before rearrangements within the spliceosome release U1 and U4 prior to the first catalytic step. This results in the formation of a downstream lariat exon and release of the upstream exon. The two exons are ligated during the second step of splicing and the intron lariat is de-branched and degraded. In higher eukaryotes, splicing is thought to occur by exon definition whereby splice sites are recognised through interactions occurring across exons rather than over the much longer introns [2]. In this model, the removal of the first and final intron involves the 5′ cap and the cleavage and polyadenylation signal, respectively [3]–[6].

Splicing is also tightly coupled to transcription by RNA polymerase II (Pol II) [7]. Several recent reports demonstrated that the majority of introns are removed co-transcriptionally before Pol II terminates transcription [8]-[12]. There is a general polarity to this process such that 5′ introns are more frequently subject to co-transcriptional splicing with some 3′ introns removed after processing at the poly(A) site [9]–[11], [13], [14]. Mechanistically, this is because 3′ end processing requires prior recognition of the terminal 3′ splice site but not removal of the intron [15]. The multiple studies showing that splicing is mostly co-transcriptional are corroborated by findings that the majority of activated spliceosomes co-purify with chromatin [16]. The active spliceosomes that are nucleoplasmic are present in speckles that also contain the splicing factor, SC35 [16].

SC35 speckles contain many factors involved in pre-mRNA processing, particularly splicing [17], [18]. It is generally accepted that Pol II is not enriched within speckles but it has been found at their periphery [19], [20]. It was also demonstrated that pre-mRNAs associate with speckles in an intron-dependent manner and that splicing could occur in these regions [21]. Consistent with an association between speckles and intron removal, small molecule inhibitors of splicing induce the appearance of enlarged nuclear speckles containing both polyadenylated RNA and SC35 [22]–[24]. Polyadenylated mRNA also accumulates in speckles following depletion of factors involved in its export [16], [21]. Indeed, splicing is required for the export of intron-containing pre-mRNA through deposition of the Exon Junction Complex (EJC) and the export factor TAP [25]–[30]. SC35 speckles therefore constitute sites of splicing factor storage, in which pre-mRNA processing and final steps in mRNP remodelling can take place prior to export into the cytoplasm.

As would be expected for such a complex and fundamental process, splicing is subject to strict nuclear quality control. This was first observed in budding yeast where mutations in either the exosome complex or Rat1 cause unspliced precursor RNAs to accumulate, with the exosome playing the major role in their degradation [31], [32]. In human cells, the Rrp6 component of the nuclear exosome as well as the Rat1 homologue Xrn2 are also involved in the quality control of transcripts when splicing is impaired, either by mutation or through treatment with Spliceostatin A (SSA) [33]–[35]. Interestingly, SSA also promotes a major increase in the level of some unspliced pre-mRNAs, which are not targeted by either Rrp6 or Xrn2 [24], [33], [36]. Instead, they have been observed to accumulate as polyadenylated species in the nucleoplasm of cells with a certain proportion leaking into the cytoplasm to be translated [22]–[24], [37]. It is not established why these transcripts are not subject to rapid nuclear degradation.

We have studied the fate of transcripts that accumulate following blocks to splicing using a morpholino (AMO) directed to U4 snRNA, which we show to inhibit splicing in a dose-dependent manner. Like small molecule splicing inhibitors, U4 AMO treatment causes polyadenylated RNA to accumulate in nuclear speckles together with SC35 and nuclear poly(A) binding protein (PABPN1). We also detect a substantial increase in the abundance of several polyadenylated pre-mRNAs in both the chromatin and nucleoplasmic fractions isolated from U4 AMO treated cells. These transcripts remain stable in the nucleus following prolongued transcription inhibition and nuclear exoribonucleases do not concentrate in speckles following splicing blocks. We show that inhibition of 3′ end cleavage and polyadenylation impairs pre-mRNA accumulation and speckle formation following splicing inhibition. Finally, we provide evidence that some unspliced pre-mRNAs, produced in cells where U4 snRNA is partially inhibited, are capable of delayed splicing. These data uncover processes ensuring the stable nuclear retention of unprocessed RNA following splicing inhibition.

## Materials and Methods

### Primers, siRNAs and Morpholinos

Intronless transcripts

c-JunFCCCCAAGATCCTGAAACAGA

c-JunRCCGTTGCTGGACTGGATTAT

GLUD2FGAATCCATGGACGCATCTCT

GLUD2RTCCCATCAGACTCACCAACA

TAF7FCTCCTCACGAACTGGAGAGC

TAF7RCCATAACACAGGGCAGGTCT

U12 intron containing transcript

FAM96BsplFATGCCAACCCCCTCATCTAC

FAM96BsplRAACCCGCACCTGCTCTACTA

FAM96BusFATGCCAACCCCCTCATCTA

FAM96BusRAGCGGCGGATATCGAAGAT

U2 intron containing transcripts

P27splFAATGCGCAGGAATAAGGAAG

P27splRATTTGGGGAACCGTCTGAA

P27usFGCTAACATACTGACAAAATAATTCCTG

P27usRCATGTATATCTTCCTTGCTTCATCA

MycsplFGAGGCTATTCTGCCCATTTG

MycsplRCACCGAGTCGTAGTCGAGGT

MycusRCTCTGACCTTTTGCCAGGAG

MycusFCCAGGCTTAGATGTGGCTCT

MycUCPA FGGCAAATATATCATTGAGCCAA

Myc UCPA RCCCAGACCCATTTCAACAGA

ETF1usFTGCAAGAGAATAGGGCTTCC

ETF1usRCGGACCCATGTCGACTACCT

ETF1splFACAGGAACGTGGAGATCTGG

ETF1splRCAGGACTGAAAGGCGGTTTA

HSPA9usFGGAGGGGGAGTGGAATAGAA

HSPA9usRAGAGCCTTCTCGCTCAGATG

HSPA9splFGCAATCAAGGGAGCAGTTGT

HSPA9splRGTCGCTCACCATCTGCTGTA

Histone

Hist1EFTTCAACATGTCCGACTGC

Hist1ERAGGCGGCAACAGCTTTAGTA

U6 snRNA

U6FACATATACTAAAATTGGAACGATAC

U6RGGAACGCTTCACGAATTTGCGT

AMOs

ControlCCTCTTACCTCAGTTACAATTTATA

U4TACGATACTGCCACTGCGCAAAGCT

U6CCATGCTAATCTTCTCTGTATCGTT

U6atacAACCTTCTCTCCTTTCATACAACAC

siRNAs

ControlLife technologies silencer negative control #1

CPSF73Life technologies silencer select s28533

### Cell culture

Cells were grown in DMEM supplemented with 10% foetal calf serum. Electroporation of AMOs (10 nmol unless otherwise stated) was performed on a confluent 10 cm diameter dish of cells in 400 µl DMEM using a 4 mm gap cuvette (960 µF, 280 v in a Biorad gene pulser). RNA was isolated three hours after electroporation unless cells were subsequently treated. For transient transfection 2–3 µg of plasmid was transfected using JetPrime (Polyplus) and experiments were performed 48 hours later. For RNAi, 20% confluent 60 mm dishes were transfected with 18 µl of 2 µM siRNA and 5 µl RNAiMAX (Life Technologies) and left for 72 hours. The transfection was repeated after re-plating of cells to 20% confluence and RNA/protein was isolated after a further 48 hours. Actinomycin D was used at a final concentration of 10 µg/ml, Cordycepin was used at 50 µg/ml for 2 hours and Pladienolide B was used at a concentration of 1 µM for 3 hours.

### Antibodies

CPSF73 (Sigma, C2747), Tubulin (Sigma, T6557), PABPN1 (Abcam, 75855), SC35 (Sigma, 4045), Dbr1 (Proteintech, 16019-1-AP), Pol II Serine 2-P (Chromotek, 3E10), Pol II H224 (Santa Cruz, sc-9001x), U2AF65 (Sigma, U4758), Anti flag (Sigma, MS2), Rrp6 (Abcam, 50558).

### RNA analysis

Total RNA was isolated using Trizol (Life Technologies) and DNase treated with Turbo DNase (Life Technologies). Isolation of chromatin-associated and nucleoplasmic RNA is described in detail elsewhere [33]. For real-time PCR analysis, 1 µg RNA was reverse transcribed using Inprom II (Promega). Parallel reactions were performed in the absence of reverse transcriptase to control for DNA contamination. 1/20^th^ of the cDNA mix was used for real-time PCR using 5–10 pmol of forward and reverse primer and Brilliant III SYBR mix (Agilent Technologies) in a Qiagen Rotorgene machine. Differences were calculated using comparative quantitation.

### Microscopy

For immunofluorescence (IF) of proteins, cells grown on cover slips were fixed with 3.7% formaldehyde (10 min) and permeabilised by incubation with 0.5% Triton X 100 in PBS (10 min). After three short washes with PBS, cells were blocked for 1 hour with 10% goat serum (Life Technologies) and incubated with primary antibody in 10% goat serum. Following three more washes, they were incubated with the secondary antibody in 10% goat serum for 1 hour. Finally, after 3 further washes with PBS cover slips were mounted (gold anti-fade, Life Technologies) and sealed. For FISH, cells were grown, fixed and permeabilised as for IF and then equilibrated with 30% Formamide in 2× SSC (2×15 min). They were then hybridised with the probe over night at 37°C in a humid chamber (30% Formamide, 0.02% BSA, 0.1 µg/ml tRNA, 10% Dextran Sulphate, 30 ng probe, 2xSSC, 0.5 µl RNase Inhibitor). Cells were washed and mounted as for IF. For 5-Ethynyl Uridine (EU) labelling, electroporated cells were incubated with 0.5 mM EU (Life technologies), washed and permeabilised as for IF. Subsequently, click ligation was performed using a Click-IT kit as per the manufacturers' guidelines (Life Technologies). Cells were mounted and visualised as for IF. All pictures were obtained using a Deltavision Elite (Applied Precision, Issaquah Washington) microscope and either a Coolsnap HQ (Photometrics UK, Marlow, Bucks) or Cascade II EMCCD (Photometrics UK, Marlow, Bucks) camera. The microscope was equipped with an x100 PlanAPO NA 1.4 (Olympus UK) objective. The deconvolution software used was Softworx (Applied Precision, Issaquah Washington).

## Results

### A U4 AMO is a potent and dose-dependent inhibitor of pre-mRNA splicing

We previously described the use of an anti-sense morpholino (AMO) directed to U4 snRNA that acts as an efficient splicing inhibitor *in vivo* [15]. Prior to spliceosome activation, U4 is extensively base-paired with U6 which sequesters the catalytic activity of the latter [15], [38]–[40]. The region of U4 that forms stem II of this interaction is crucial for splicing *in vitro* [41], [42]. Our U4 AMO is designed to block the interaction between U4 and U6 by preventing the formation of stem II and therefore inhibiting the production of active spliceosomes. In so doing, pre-mRNAs can assemble U1 and U2 complexes but splicing is blocked prior to catalysis [15], [42]. The use of AMOs to inhibit splicing is advantageous over protein knock-down by RNAi because they act much more rapidly (3 hours versus typically 72 hours), somewhat limiting off-target effects.

**Figure 1:**
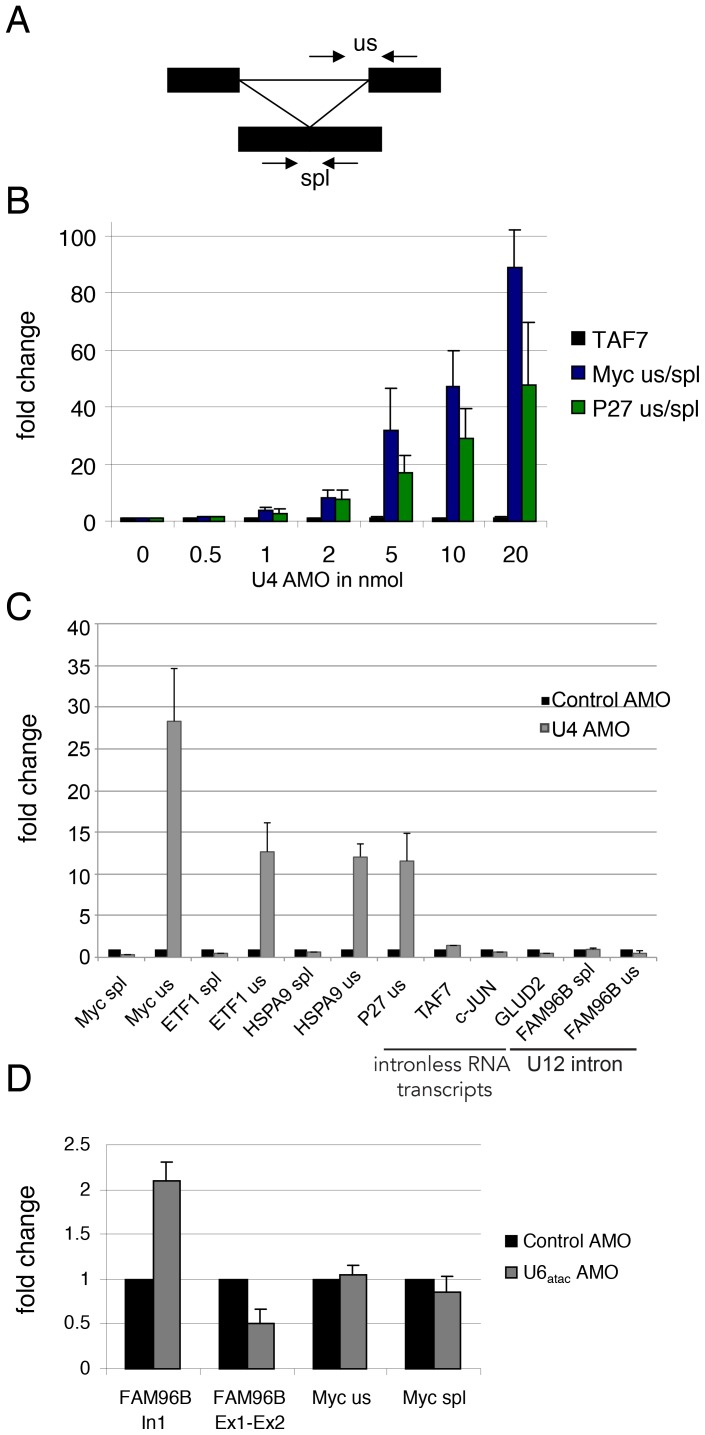
A. Diagram depicting primer pairs used to analyse spliced (spl) and unspliced (us) pre-mRNA. Exons are black boxes. B. Quantitation of P27 and Myc splicing in cells treated with the indicated amounts of U4 AMO. Splicing inhibition was calculated as a ratio of signal for unspliced versus spliced RNA and expressed as a fold change compared to cells treated with control AMO (0 nmol U4 AMO). Intronless TAF7 transcripts were analysed as a control. C. Quantitation of unspliced P27, Myc, HSPA9 and ETF1 pre-mRNA, spliced Myc, HSPA9 and ETF1 mRNA, intronless c-Jun, TAF7 and GLUD2 and minor spliceosome-dependent FAM96B splicing in cells treated with control or 10 nmol U4 AMOs. Results are expressed as a fold change over values obtained in control cells after normalising to Histone H1E levels. D. Quantitation of FAM96B and Myc splicing in cells treated with control or U6atac AMOs. Values are expressed as a fold change over values obtained in control cells after normalising to Histone H1E levels.

We wished to use this reagent to study the consequences of splicing inhibition *in vivo*. Although we have previously demonstrated its effectiveness as a splicing inhibitor at the RNA level [15], we had not studied the fate of the resulting pre-mRNAs. To begin with, we assessed the dose-dependence of its effect on intron removal. HeLa cells were electroporated with increasing amounts of U4 AMO (0.5–20 nmol) or 10 nmol of control AMO (0 nmol U4 AMO). Total RNA was then isolated after 3 hours to limit off-target effects. Following reverse transcription with random hexamers, real-time PCR was performed using primers that detect intron-containing and spliced P27 or Myc transcripts (Figure 1A). We chose these transcripts as they accumulate markedly in cells where splicing is inhibited [15], [24], [33]. Splicing was then plotted as a ratio of unspliced versus spliced product (Figure 1B). Significant splicing impairment was observed with 1 nmol AMO (∼4-fold); however inhibition was progressively more substantial with 20 nmol U4 AMO inducing a 50–100 fold inhibition of splicing for both of these transcripts. Thus, the U4 AMO is a powerful inhibitor of splicing that acts in a dose-dependent manner.

Next, we assessed the effect of U4 AMO treatment on a range of transcripts additional to those from *MYC* and *P27* genes (Figure 1C). To this end, total RNA was isolated from cells treated with 10 nmol of control or U4 AMO and reverse transcribed with random hexamers. Since *P27* and *MYC* genes are relatively short and contain only two introns, we measured spliced and unspliced transcripts from two longer genes that contain multiples introns: *ETF1* and *HSPA9*. To verify the specificity of U4 AMO treatment towards U2-dependent splicing, transcripts from three intron-less genes (*TAF7*, *c-JUN* and *GLUD2*) and a splicing event performed by the minor U12-dependent spliceosome (that of FAM96B pre-mRNA intron 1) was assayed. As expected, a substantial increase in the level of unspliced Myc and P27 RNA was again evident with a reduction in spliced Myc also observed. Similar effects were observed for ETF1 and HSPA9 transcripts. However, the levels of intron-less transcripts were unaffected and FAM96B intron 1 showed no evidence of increased retention. These data strongly suggest that the U4 AMO affects the splicing of transcripts that are substrates for the U2-dependent spliceosome but not the level of intron-less or minor-spliceosome dependent RNAs.

As a final specificity control for the AMO approach, we elected to inhibit the minor spliceosomal U6atac snRNA. We treated cells with a control AMO or an AMO directed to the U6atac snRNA [43]. Total RNA was again reverse transcribed with random hexamers and real-time PCR was used to detect spliced and unspliced Myc transcripts as well as to assess splicing of FAM96B intron 1 (Figure 1D). The U6atac AMO did not affect Myc pre-mRNA splicing, which was expected since its splicing depends on the major spliceosome. However, it caused an accumulation of FAM96B intron 1 with a concomitant reduction in the corresponding spliced product. We conclude that the U4 AMO inhibits splicing by the major spliceosome and the U6atac AMO inhibits the minor splicing pathway. These data highlight the specificity and potency of the U4 AMO-based approach to inhibit splicing.

### Splicing inhibition induces the formation of enlarged nuclear speckles containing polyadenylated RNA

**Figure 2:**
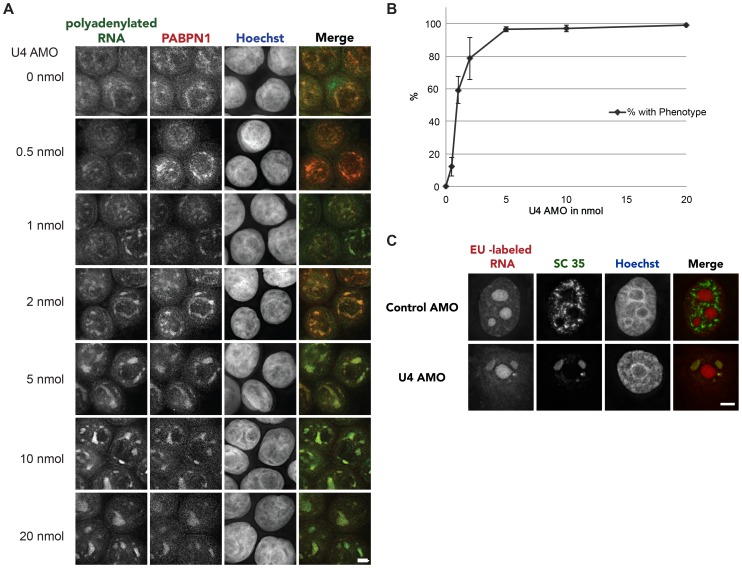
A. FISH and IF to detect polyadenylated RNA or PABPN1 respectively in cells electroporated with control AMO or the indicated amounts of U4 AMO. Scale bar is 5 µm. B. Graph showing the percentage of cells displaying an enlarged speckle phenotype under conditions of increased U4 AMO concentrations. 100 cells were counted per condition over three separate experiments. C. EU labelling of nascent RNA and IF of SC35 in cells treated with control or 10 nmol U4 AMO. Scale bar is 5 µm.

Previous studies demonstrated that treating cells with the small molecule splicing inhibitors Meayamycin (MY) and Spliceostatin A (SSA) induced the accumulation of polyadenylated RNA in enlarged nuclear speckles [22]–[24]. These compounds inhibit splicing after U1 and U2 snRNAs are recruited to the intron and specifically target the SF3b complex [24], [44]–[47]. We were interested to see whether inhibition of U4 snRNA might cause a similar enlargement of nuclear speckles even though SF3b would not be directly targeted. To test this, we electroporated cells with increasing amounts of U4 AMO or 10 nmol of control AMO (0 nmol U4 AMO) and tested the location of polyadenylated RNA and the nuclear poly(A) binding protein, PABPN1, by fluorescence in situ hybridisation (FISH) and immunofluorescence (IF) respectively (Figure 2A). Similar to MY and SSA, treatment of cells with the U4 AMO caused polyadenylated RNA to accumulate in enlarged nuclear speckles. The same was true for PABPN1. As the concentration of AMO was increased, more cells displayed this phenotype confirming its dose-dependence (Figure 2B). Finally, Pladienolide B (PB), an established SF3b inhibitor, gave the same result as did an AMO directed to U6 snRNA (Figure S1).

We next wished to determine if this polyadenylated RNA represented a major or a minor fraction of transcripts that were redistributed following splicing inhibition. To do this, cells were electroporated with control or U4 AMOs and growing cells were subjected to metabolic labelling using 5-Ethynyl Uridine (EU). EU will detect all synthesised transcripts rather than only polyadenylated RNA. Cells were subsequently labelled with Alexa Fluor 594 by a click reaction and visualised (Figure 2C). In both control and U4 AMO treated cells, the majority of signal was present in nucleoli consistent with the high level of rRNA transcription that occurs in cells. However, there was an additional concentration of EU RNA in enlarged speckles outside of the nucleolar region in U4 AMO treated cells but not in control cells. Importantly, these enlarged regions overlapped with that of the well characterised speckle marker SC35, as determined by IF. Together, the FISH and IF data in Figure 2 show that polyadenylated RNA accumulates in enlarged PABPN1 and SC35-containing nuclear speckles when splicing is inhibited by a U4 AMO. The fact that U4 AMO specifically inhibits U2-dependent splicing suggests that some of the RNA within speckles is pre-mRNA. Indeed, an AMO against U6atac snRNA did not induce this phenotype presumably due to the small number of splicing events that are inhibited (Figure S2).

### Enlarged speckles contain U2AF65 but not active RNA polymerase II

**Figure 3:**
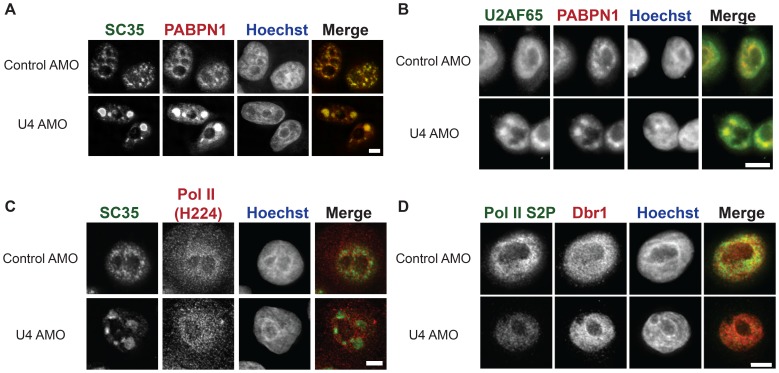
A. IF of SC35 and PABPN1 in cells treated with control or U4 AMO. Scale bar is 5 µm. B. IF of PABPN1 and U2AF65 in cells treated with control of U4 AMO. Scale bar is 20 µm. C. IF of total Pol II (H224) and SC35 in cells treated with control or U4 AMO. Scale bar is 5 µm. D. IF of Dbr1 and Pol II phosphorylated on Serine 2 (Pol II S2P) in cells treated with control of U4 AMO. Scale bar is 5 µm.

To characterise the enlarged nuclear speckles further, we performed a limited IF analysis of additional factors to test their re-localisation, if any, following splicing inhibition by the U4 AMO. We first confirmed that PABPN1 and SC35 were co-localised in enlarged speckles after U4 AMO treatment (Figure 3A). Next, we assayed the U2AF65 splicing factor (Figure 3B, Figure S3A). U2AF65 became concentrated in enlarged nuclear speckles together with PABPN1 following U4 AMO treatment. This is consistent with the expectation that U4 AMO blocks splicing following U1 and U2 snRNA recruitment.

We next sought to determine whether Pol II, or factors that would be expected to act after U4 snRNA in the splicing process, would accumulate in enlarged speckles following splicing inhibition. We first performed IF on total Pol II (H224) (Figure 3C, Figure S3B). Signal was observed throughout the cell with a noticeable enrichment within the nucleus. However, contrary to SC35, it was not dramatically re-localised following U4 AMO treatment suggesting that enlarged speckles do not represent sites of transcription. Next, we analysed transcriptionally active Pol II phosphorylated on Serine 2 of its C-terminal domain (Pol II S2P) as well as the debranching enzyme Dbr1, which is required after U4 function in splicing (Figure 3D, Figure S3C). Although U4 AMO caused a reduction in Pol II S2P signal, it did not promote its localisation to enlarged speckles. This again suggests that there is little or no transcription within the nuclear speckles and that therefore the RNA within them is not nascent. Similarly, Dbr1 was also broadly distributed within the nucleus but did not localise to nuclear speckles following U4 AMO treatment. This is consistent with the fact that it functions subsequently to U4 snRNA in the splicing process. Thus, enlarged nuclear speckles observed following U4 inhibition contain proteins that act prior to U4 snRNA in splicing, as well as PABPN1 presumably due to the polyadenylated nature of the transcripts. However, Dbr1 and Pol II are not concentrated within the enlarged speckles.

### U4 AMO treatment causes the accumulation of polyadenylated pre-mRNA in chromatin and nucleoplasmic RNA fractions

**Figure 4:**
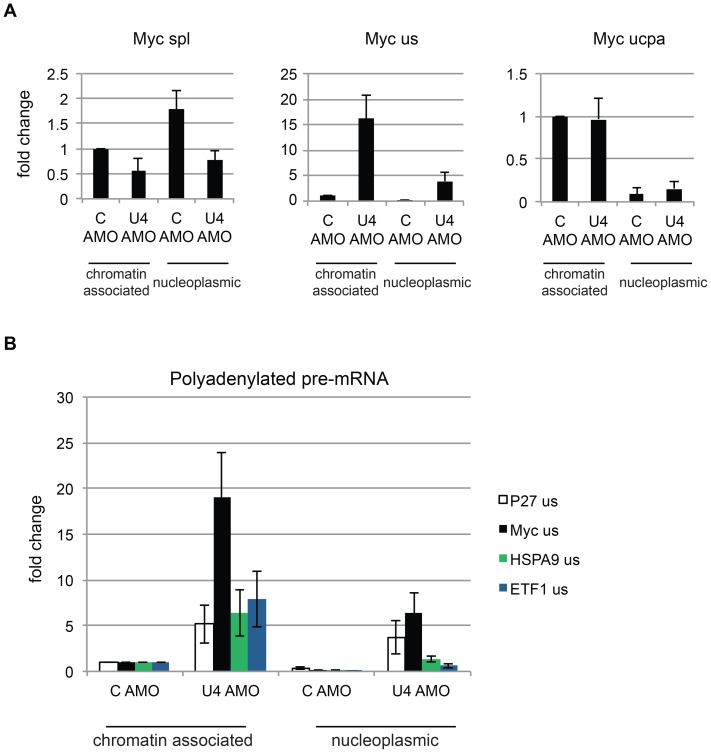
A. Isolation of chromatin-associated and nucleoplasmic RNA from cells treated with control or U4 AMO. Spliced (spl), unspliced (us) and non-pA cleaved (ucpa) Myc transcripts are detected. Following normalisation to intronless c-Jun RNA, values are plotted as a fold change compared to the chromatin-associated fraction of control cells (given a relative value of 1). B. Isolation of chromatin-associated and nucleoplasmic RNA from cells treated with control or U4 AMO. Unspliced and polyadenylated Myc, P27, HSPA9 and ETF1 transcripts are detected. Following normalisation to intronless c-Jun RNA, values are plotted as a fold change compared to the chromatin-associated fraction of control cells (given a relative value of 1).

Data so far show that U4 AMO inhibits splicing and causes polyadenylated RNA to accumulate in enlarged nuclear speckles. To quantitate the effects of U4 AMO on pre-mRNA levels and their distribution within the nucleus, we used a well-characterised method to biochemically isolate chromatin-associated and nucleoplasmic transcripts from nuclei [48]. This was done in cells treated with control or U4 AMOs and isolated RNA was reverse transcribed with random hexamers. To verify efficient separation of the two fractions, we monitored the level of spliced, unspliced and non-pA cleaved Myc RNA (Figure 4A). As previously observed [9], [12], [33], [49], spliced transcripts were more enriched in the nucleoplasm compared to chromatin and were depleted in samples from U4 AMO treated cells. Unspliced RNA was predominantly chromatin-associated in control cells in agreement with the view that pre-mRNA processing is normally coupled to transcription. An increase in unspliced RNA was seen in both chromatin-associated and nucleoplasmic fractions following U4 treatment as expected. This is similar to previous findings from our laboratory and suggests that while some unspliced transcripts are retained on chromatin following splicing inhibition, a proportion is released [15]. Finally, non-pA cleaved RNA was predominantly chromatin-associated in both control and U4 treated cells and was unaffected by splicing inhibition. As well as being consistent with our previous finding that poly(A) site cleavage is unaffected by U4 inhibition [15], this provides evidence that unspliced transcripts accumulating in the nucleoplasm are cleaved at their poly(A) site.

Accordingly, we next assayed the distribution of polyadenylated unspliced RNA within nuclei of control and U4 AMO treated cells. Chromatin-associated and nucleoplasmic RNA from these cells was reverse transcribed with oligo-dT before detection of unspliced P27, Myc, ETF1 and HSPA9 transcripts by real time PCR (Figure 4B). Unspliced, polyadenylated pre-mRNA was increased in both fractions in U4 AMO treated samples as compared to those isolated from control cells. This confirms that some unspliced pre-mRNA is polyadenylated. Furthermore, the presence of these species in the nucleoplasm following splicing inhibition is consistent with the appearance of enlarged speckles enriched in polyadenylated RNA under the same conditions. Finally, transcripts from the P27 and Myc genes accumulated more markedly in the nucleoplasmic fraction than those from the longer HSPA9 and ETF1 genes following U4 AMO treatment. This may suggest transcript-specific regulation regarding the release of unspliced pre-mRNA from chromatin.

### Nuclear degradation machinery does not concentrate in speckles following splicing inhibition

**Figure 5:**
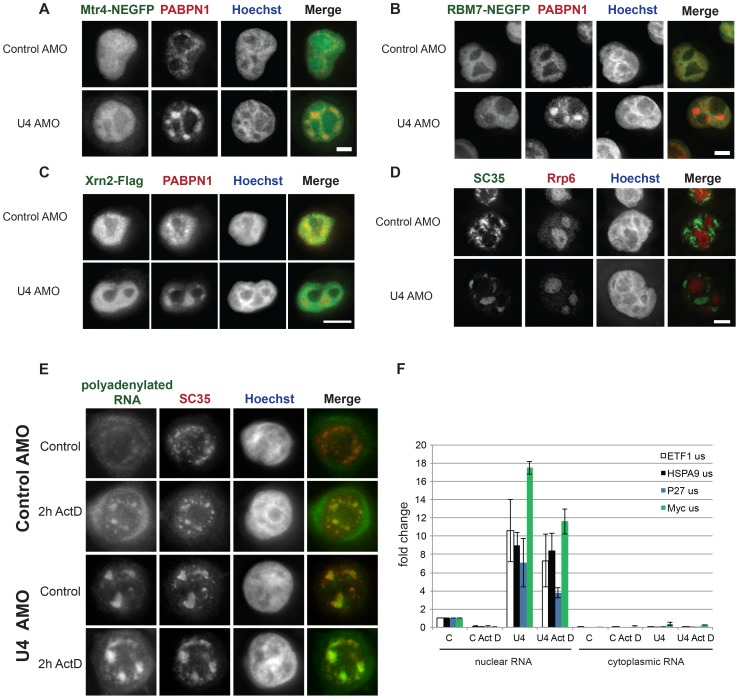
A. IF of EGFP-Mtr4 and PABPN1 in cells treated with control or U4 AMO. Scale bar is 5 µm. B. IF of EGFP-Rbm7 and PABPN1 in cells treated with control or U4 AMO. Scale bar is 5 µm. C. IF of Flag-Xrn2 and PABPN1 in cells treated with control or U4 AMO. Scale bar is 15 µm. D. IF of Rrp6 and SC35 in cells treated with control or U4 AMO. Scale bar is 5 µm. E. FISH and IF to detect polyadenylated RNA and SC35 in cells treated with control or U4 AMO followed by 2 hours in the presence of Act D or ethanol (Control). Scale bar is 15 µm. F. Real-time PCR analysis of polyadenylated and unspliced Myc, P27, HSPA9 and ETF1 transcripts in the nuclear and cytoplasmic fractions of cells electroporated with control (C) and U4 AMO and subsequently treated with Act D or ethanol (use of Act D is indicated below x-axis). Quantitation is shown as fold change compared to amounts found in the nuclei of control AMO treated cells after ethanol treatment, which were given a value of 1.

The accumulation of pre-mRNA transcripts following U4 AMO treatment is dramatic suggesting that they are not rapidly degraded. Indeed, we have previously shown that following SSA treatment, some pre-mRNAs accumulate and are not affected by depletion of nuclear exoribonucleases suggesting that they are not degraded despite their inability to be spliced [33]. To gain more understanding of the resistance of these unspliced transcripts to degradation, we analysed the cellular distribution of major nuclear RNA decay factors in control and U4 AMO treated cells. We performed IF following over-expression of GFP-tagged core nuclear exosome component Mtr4 in control and U4 AMO treated cells (Figure 5A, Figure S4A). This protein was found throughout the nucleus, including nucleoli where it was previously observed [50]. The more homogeneous nuclear signal that we observed might be due to the fixation of cells necessary for this co-localisation experiment or electroporation of AMOs [51]. However, while U4 AMO treatment caused accumulation of PABPN1 in enlarged speckles there was no similar concentration of GFP-tagged Mtr4 in the same cells. A similar result was obtained with GFP-tagged Rbm7, a nuclear exosome co-factor that does not localise to nucleoli [50] (Figure 5B, Figure S4B). We also tested the effect of U4 AMO on the localisation of over-expressed Xrn2, which is the major nuclear 5′→3′ exonuclease. While PABPN1 was concentrated in enlarged speckles following U4 AMO treatment, Xrn2 did not (Figure 5C, Figure S4C). These data indicate that over-expressed tagged exonucleases do not concentrate in nuclear speckles when splicing is inhibited under these experimental conditions.

Because the above results were obtained using tagged and over-expressed factors, we asked whether the localisation of an endogenous exoribonuclease was affected by U4 AMO treatment (Figure 5D, Figure S4D). We analysed the Rrp6 component of the nuclear exosome, which has a well characterised role in pre-mRNA quality control in budding yeast and humans [31], [35], [52]. Moreover, Rrp6 is involved at the interface between polyadenylation and degradation [52]–[54]. In control AMO treated cells, Rrp6 was predominantly localised to nucleoli as previously reported and consistent with its function in rRNA processing [50], [55], [56]. This pattern was essentially unchanged following U4 AMO treatment showing that Rrp6 does not re-localise to enlarged nuclear speckles following splicing inhibition. In contrast, SC35 was localised to enlarged speckles in the same samples. These data indicate that exonucleases do not re-localise to enlarged nuclear speckles following U4 AMO treatment. It is consistent with the observation in Figure 4 that pre-mRNAs accumulate in the nucleoplasm as well as the chromatin following U4 AMO treatment.

### Unspliced pre-mRNA is stable within the nucleus following transcription inhibition

Some leakage of unspliced transcripts into the cytoplasm occurs in SSA treated cells [24], [37]. However, it is not clear if transcripts that are observed in speckles can eventually be exported or not. To test this, we performed FISH and IF on polyadenylated RNA and SC35, respectively, using cells electroporated with control or U4 AMO. Following this, cells were treated or not for two hours with the transcriptional inhibitor, Actinomycin D (Act D). As can be seen in Figure 5E, U4 AMO treatment caused polyadenylated RNA and SC35 to accumulate in enlarged speckles as expected. However, this was still the case after Act D treatment suggesting that bulk polyadenylated RNA is nuclear-restricted following U4 AMO treatment rather than exported or degraded over the period of transcription inhibition.

FISH and IF signal within enlarged speckles were sometimes more diffuse after Act D treatment, which potentially complicates the interpretation of these experiments (Figure S4E). Therefore, we directly analysed individual pre-mRNA transcripts in nuclear and cytoplasmic RNA fractions from control and U4 AMO treated subsequently treated with Act D or, as a control, its ethanol solvent. Following reverse transcription with oligo-dT, RNA was real-time PCR amplified to detect unspliced Myc, P27, ETF1 and HSPA9 transcripts (Figure 5F). In cells treated with control AMO these were mainly nuclear and Act D treatment caused their depletion confirming that transcription was inhibited. In U4 AMO treated cells, unspliced pre-mRNA accumulated in the nucleus as expected; however, after Act D treatment the majority of this signal remained nuclear. This suggests that a large proportion of polyadenylated pre-mRNA that accumulates in the nucleus following U4 AMO treatment is retained there stably, at least under the conditions used here.

### Enlarged speckles do not form when cleavage and polyadenylation are inhibited

**Figure 6:**
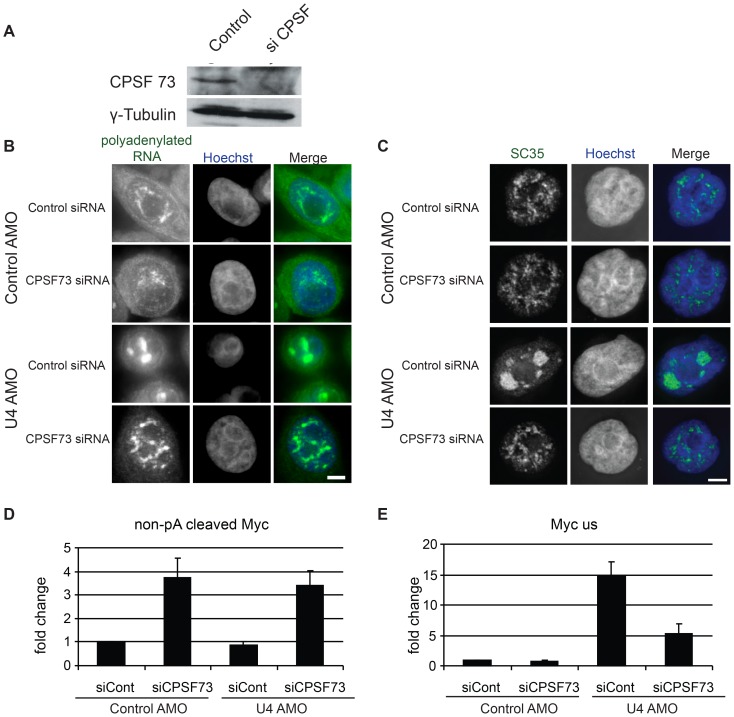
A. Western blotting of CPSF73 (top panel) and Tubulin (lower panel) in cells treated with control or CPSF73 siRNAs. B. FISH analysis of polyadenylated RNA in cells treated with control or CPSF73 siRNAs and, subsequently, with control or U4 AMO. Scale bar is 5 µm. C. IF of SC35 in cells treated with control or CPSF73 siRNAs and, subsequently, with control or U4 AMO. Scale bar is 5 µm. D. Quantitation of non-poly(A) site cleaved Myc transcripts in cells treated with control or CPSF73 siRNAs and, subsequently, with control or U4 AMO. Values are expressed as a fold change compared to control siRNA transfected cells treated with control AMO after normalising to U6 snRNA. E. Quantitation of unspliced Myc transcripts (intron 2-exon3) in cells treated with control or CPSF73 siRNAs and, subsequently, with control or U4 AMO. Values are expressed as a fold change compared to control siRNA transfected cells treated with control AMO after normalising to U6 snRNA.

Data so far suggest that inhibition of U4 snRNA causes accumulation of unspliced, polyadenylated pre-mRNA in the nucleus and the appearance of enlarged nuclear speckles. We next wanted to address the mechanistic basis for these two observations and began by investigating the role of 3′ end processing since poly(A) site cleavage is well known to be required to both release RNA from chromatin and stabilise it [57], [58]. We depleted the 3′ end processing endonuclease, CPSF73, from HeLa cells (Figure 6A). Cells were next treated with control or CPSF73 specific siRNAs and then electroporated with control or U4 AMOs before visualisation of polyadenylated RNA by FISH. In control siRNA treated cells, the U4 AMO induced accumulation of these transcripts in speckles as observed before (Figure 6B). However, U4 AMO treatment of CPSF73 depleted cells did not have this effect and speckles were smaller and more numerous.

It might be expected that polyadenylated RNA signals would be diminished following CPSF73 depletion due to disruption of poly(A) site processing. Therefore we also monitored speckle formation using IF to detect SC35 in control and CPSF73 depleted cells treated with control or U4 AMOs (Figure 6C, Figure S5A). As before, U4 AMO treatment of control cells induced the accumulation of SC35 in enlarged speckles. However, when CPSF73 depleted cells were treated with the U4 AMO, SC35 did not show this dramatic re-localisation. This experiment suggests that the formation of enlarged nuclear speckles in U4 AMO treated cells requires CPSF73 activity. The formation of enlarged nuclear speckles was similarly abrogated by inhibiting polyadenylation (Figure S5B and S5C).

The above experiments suggest that cleavage and polyadenylation are critical for the formation of enlarged nuclear speckles. Given the confirmed stabilising role for a poly(A) tail we also wondered whether CPSF73 depletion might impair the general accumulation of unspliced pre-mRNA following U4 AMO treatment. To test this, we isolated total RNA from both control and CPSF73 depleted cells treated with control or U4 AMO (Figure 6D). cDNA was then generated with random hexamers. To confirm that CPSF73 depletion was sufficient to impair poly(A) site cleavage we used primers spanning the Myc poly(A) site to detect transcripts on which 3′ end processing had not occurred. 3–4 fold more of this transcript was recovered in samples from CPSF73 depleted cells indicating that knock-down was sufficient to disrupt cleavage and polyadenylation. This was the case in both control and U4 AMO treated cells indicating that splicing inhibition does not influence the function of CPSF73 in poly(A) site cleavage.

Next we monitored the accumulation of unspliced Myc transcripts in all four samples (Figure 6E). In cells treated with control siRNA, U4 AMO caused a large accumulation of this species as expected. However, fewer unspliced RNAs accumulated in CPSF73 depleted cells treated with U4 AMO indicating that cleavage and polyadenylation stabilises unspliced RNA. A requirement for cleavage and polyadenylation for speckle formation and pre-mRNA accumulation may be explained by 3′ end processing being necessary to release RNA from chromatin into speckle regions. However, knock-down of cleavage and polyadenylation may reduce transcription and pre-mRNA stability more generally thus reducing the detectable poly(A)+ FISH signal as well as the accumulation of selected pre-mRNAs such as Myc.

### Transcripts retain the ability to be spliced following U4 snRNA inhibition

We have shown that, following splicing inhibition, many pre-mRNAs accumulate as polyadenylated transcripts in a manner dependent on cleavage and polyadenylation. Although unprocessed, these transcripts are not degraded. Recent work indicates that some splicing occurs after 3′ end processing and transcription are completed [14], [16]. Moreover, it has been proposed that some of this splicing occurs in nuclear speckle domains [16], [21]. We wondered whether transcripts that accumulate following U4 AMO treatment might somehow resemble processing intermediates that can go on to be spliced, which might explain why they are not degraded. If this is the case then transcripts whose splicing is initially inhibited under these conditions might be capable of being spliced in a delayed manner.

**Figure 7:**
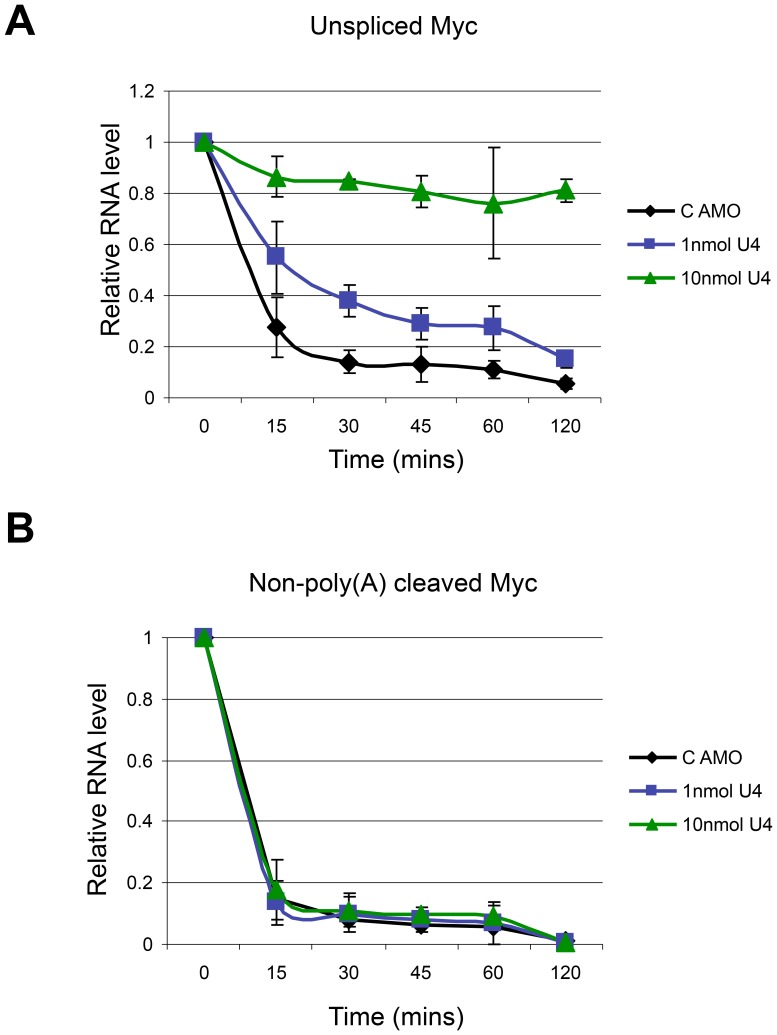
A. Act D time course analysis of unspliced Myc RNA (intron 2-exon 3) in cells treated with control AMO or 1 and 10 nmol U4 AMO. For each condition, values are presented relative to levels present at time zero (given a value of 1). B. Act D time course analysis of non-pA cleaved Myc RNA in cells treated with control AMO or 1 and 10 nmol U4 AMO. For each condition, values are presented relative to levels present at time zero (given a value of 1).

Unfortunately, AMOs and small molecule inhibitors of splicing are not readily reversible somewhat confounding this analysis. However, results in Figure 1 show that lower concentrations of U4 AMO inhibit splicing but in an incomplete manner. Results in Figure 2 confirm that these concentrations are also sufficient to induce the appearance of enlarged speckles containing polyadenylated RNA. This being the case, we wanted to establish whether or not the unspliced transcripts that accumulate under these conditions can still be spliced. Accordingly, we monitored the levels of unspliced Myc transcripts (intron 2-exon 3) following transcriptional inhibition by Act D (Figure 7A). We previously showed that this species is rapidly depleted following Act D treatment but not when U4 AMO was used, which confirms that its loss depends on functional splicing [15], [59]. We performed this experiment on cells treated with control AMO or with 1 or 10 nmol of U4 AMO. Levels of Myc intron 2-exon 3 RNA were determined by real-time PCR following reverse transcription. In the control treated cells, this species was strongly reduced after 15 minutes of Act D treatment. As expected, this was due to functional splicing because little reduction was observed in cells treated with 10 nmol U4 AMO even after 2 hours. This observation also highlights the stability of unspliced Myc following U4 inhibition. In cells treated with 1 nmol of U4 AMO, much of this species was depleted over the full time-course albeit at a slower rate than in control cells. These data suggest that some delayed splicing occurs in cells where U4 is partially inhibited.

It was next important to determine whether this delayed splicing occurred after cleavage at the poly(A) site, which we have shown to be required for optimal accumulation of unspliced pre-mRNA following U4 AMO treatment. To test this, the Act D time course was repeated to determine the rate of Myc poly(A) site cleavage. Primers were used that span the Myc poly(A) site such that only unprocessed transcripts were detected. Importantly, poly(A) site cleavage precludes the detection of this species and so its loss is indicative of processing (Figure 7B and [59]). As we had observed previously, this species was lost equally rapidly in control cells and cells treated with 10 nmol U4 AMO showing that intron removal is not necessary for poly(A) site processing [15]. Treatment with 1 nmol U4 AMO gave a similar result whereby poly(A) site cleavage occurred at the control rate. In sum, when compared to control AMO treatment, splicing is substantially delayed relative to poly(A) site cleavage in cells treated with 1 nmol U4 AMO. These data argue that transcripts, for which splicing is initially prevented by partial U4 AMO treatment, can still be spliced. This is likely to occur after cleavage and polyadenylation, the rate of which is unaffected by U4 inhibition. Similarly, it was shown that depletion of the CDC5L splicing factor caused accumulation of polyadenylated RNA within nuclear speckles [16]. However, reintroduction of the CDC5L protein relieved this effect. These findings suggest that some pre-mRNA transcripts that accumulate in the nucleus following splicing inhibition are in a spliceable state, which may explain their stability.

## Discussion

Here we have explored the fate of transcripts produced in cells where splicing is inhibited using a U4 AMO. Pre-mRNAs accumulate as polyadenylated species coincident with the formation of enlarged nuclear speckles containing polyadenylated RNA. Both the pre-mRNAs and speckle structures are stable and remain present in nuclei following prolongued inhibition of transcription by Act D. Consistent with their stability, we observe no re-localisation of nuclear exonucleases within the nuclei of cells when splicing is inhibited. Both pre-mRNA accumulation and speckle formation were sensitive to inhibition of pre-mRNA cleavage and polyadenylation arguing that 3′ end processing plays an important role in both events. We suggest that transcripts in cells defective for splicing are stable at least in part because they remain splice-competent. Consistently, unspliced transcripts that accumulate when splicing is only partially inhibited can be spliced in a delayed fashion.

An enlarged speckle phenotype like the one we see is also observed when cells are treated with small molecule inhibitors of splicing [22]–[24]. At least some of these transcripts are exported to the cytoplasm because their aberrant protein products are detectable [24]. Therefore, it is possible that the nonsense-mediated decay pathway is the mechanism by which they are eventually degraded. However, we observe high levels of polyadenylated RNA in the nucleus following U4 AMO treatment. Furthermore, this remains the case even after transcription is inhibited. We therefore favour the interpretation that some pre-mRNA is exported into the cytoplasm but a substantial fraction is retained in the nucleus. This is consistent with data demonstrating a strong link between splicing and mRNA export [25], [29], [30].

We were unable to unequivocally localise an individual pre-mRNA to the enlarged speckles using FISH (data not shown) even though these domains were enriched in polyadenylated RNA. Data in figure 4 support the idea of polyadenylated pre-mRNA being part of the enlarged speckles by showing their accumulation in nucleoplasmic RNA fractions from U4 AMO treated cells. Our observation that only substrates for U2-dependent splicing are up-regulated by U4 AMO treatment and that enlarged speckles only form when splicing is inhibited also suggests that speckles contain pre-mRNA. Moreover, the sensitivity of enlarged speckle formation to CPSF73 depletion suggests the presence of pre-mRNAs there since the major function of this factor is in processing poly(A) sites. Finally, others were able to localise β-globin pre-mRNA to speckles when small molecules were used to inhibit splicing – albeit for a longer period of time than we used [22], [23]. Thus, although we cannot exclude the presence of other polyadenylated RNAs in enlarged speckles, we favour the view that a proportion of RNA within them is pre-mRNA.

U4 AMO promotes the accumulation of pre-mRNA on chromatin as well as in the nucleoplasm, coincident with the appearance of enlarged speckles. This indicates two potential ways that ultimately prevent the expression of unprocessed transcripts: one that prevents their release from chromatin and another that sequesters released transcripts within nucleoplasmic domains. Interestingly, the proportion of pre-mRNA that is present in the nucleoplasm following splicing inhibition differs among the transcripts that we analysed being much higher for P27 and Myc transcripts as compared to HSPA9 and ETF1. Further studies will be required to determine the basis of this observation but it may well reflect transcript-specific requirements for chromatin release. However, it is generally the case that the majority of pre-mRNA is retained stably in the nucleus following U4 AMO treatment. We would speculate that this is due to factors that remain bound as a result of intron retention as well as the absence of proteins that are normally deposited by splicing. The former may include members of the U1 and U2 snRNPs, which remain bound in cells treated with U4 AMO [15]. Indeed U1-70k and U2AF65 are actively involved in pre-mRNA retention within the nucleus [37]. The latter class of factors is likely to include EJC components, which are bound following splicing and are required for RNA export [29], [60], [61].

As well as being retained in the nucleus due to defects in the normal pathway of mRNA biogenesis, unspliced pre-mRNAs accumulating following U4 inhibition could be subject to active quality control. Indeed, a variety of different quality control pathways have been identified that promote turnover of unprocessed or splice-defective RNA. However, the transcripts that we have analysed following U4 inhibition are stable. We speculate that this may be because they are recognised as being genuine splicing precursors rather than aberrant pre-mRNAs. We propose that early steps in spliceosome assembly must still take place in the presence of U4 AMO because recognition of the terminal 3′ splice site is required for 3′ end processing, which still occurs when U4 is inhibited [3], [15], [62]. The fact that some delayed splicing may take place when U4 is partially inhibited indicates that these early assembly steps might be sufficient to license intron removal. Furthermore, this observation supports the possibility that there may not be a fixed window in which splicing must occur. As such, when splicing is inhibited, unspliced pre-mRNAs may be released from the transcription site and accumulate in speckles or elsewhere where, under normal circumstances, splicing would be completed.

We have described mechanisms that prevent the export and expression of pre-mRNA in cells where splicing is inhibited by a U4 AMO. Rather than being degraded, these transcripts are very stable and we provide evidence that they can be spliced at a later time than normal. This pathway thus serves two useful purposes: firstly, unprocessed transcripts are prevented from being exported and secondly, transcripts that appear capable of being processed are preserved such that they may later complete their nuclear maturation.

## Supporting Information

Figure S1 **Pladienolide B or U6 AMO treatment results in the accumulation of polyadenylated RNA within enlarged nuclear speckles.** A. IF of SC35 and PABPN1 in cells treated with DMSO or the splicing inhibitor Pladienolide B (PB). Scale bar is 20 µm. B. FISH and IF of polyadenylated RNA and SC35 respectively in cells treated with DMSO or the splicing inhibitor PB. Scale bar is 20 µm. C. FISH and IF of polyadenylated RNA and SC35 respectively in cells treated with control or U6 AMO (10 nmol). Scale bar is 5 µm.
(TIFF)

Figure S2 **U6atac inhibition does not result in enlarged nuclear speckles.** A. IF of SC35 and PABPN1 in cells treated with control or U6atac AMO (10 nmol). Scale bar is 15 µm. B. FISH and IF of polyadenylated RNA and SC35 respectively in cells treated with control or U6atac AMO (10 nmol). Scale bar is 15 µm.
(TIFF)

Figure S3 **Alternative cell pictures accompanying main text** **figure 3** **.** A. IF of U2AF65 and PABPN1 in cells treated with control of U4 AMO (10 nmol). Two panels are shown per condition. Scale bar is 20 µm. B. IF of SC35 and total Pol II (H224) in cells treated with control of U4 AMO (10 nmol). Two panels are shown per condition. Scale bar is 5 µm. C. IF of Pol II S2P and Dbr1 in cells treated with control of U4 AMO (10 nmol). Two panels are shown per condition. Scale bar is 5 µm.
(TIF)

Figure S4 **Alternative cell pictures accompanying main text** **figure 5** **.** A. IF of GFP-tagged Mtr4 and PABPN1 in cells treated with control of U4 AMO (10 nmol). Two panels are shown per condition. Scale bar is 5 µm. B. IF of GFP-tagged Rbm7 and PABPN1 in cells treated with control of U4 AMO (10 nmol). Two panels are shown per condition. Scale bar is 5 µm. C. IF of flag-Xrn2 and PABPN1 in cells treated with control of U4 AMO (10 nmol). Two panels are shown per condition. Scale bar is 15 µm. D. IF of SC35 and Rrp6 in cells treated with control of U4 AMO (10 nmol). Two panels are shown per condition. Scale bar is 5 µm. E. Poly(A)+ RNA FISH and SC35 IF in control (left-hand panels) and U4 AMO treated (right-hand panels) cells treated with ethanol (control) or Act D for two hours in cells treated with control of U4 AMO (10 nmol). Two panels are shown per condition. Scale bar is 15 µm.
(TIF)

Figure S5 **pre-mRNA cleavage and polyadenylation are required for the formation of enlarged speckles following splicing inhibition.** A. Alternative pictures accompanying main text figure 6C: IF of SC35 in cells treated with control or CPSF73 siRNAs and, subsequently, with control or U4 AMO. Two data panels are shown. Scale bar is 5 µm. B. Poly(A)+ RNA FISH in control and U4 AMO treated cells treated with DMSO or the polyadenylation inhibitor cordycepin (CDY). CDY prevents the formation of enlarged poly(A)+ speckles following U4 AMO treatment. Scale bar is 5 µm. C. SC35 IF in control and U4 AMO treated cells treated with DMSO or the polyadenylation inhibitor cordycepin (CDY). CDY prevents the formation of enlarged SC35-containing speckles following U4 AMO treatment. Scale bar is 20 µm.
(TIF)
