# Supplementary figures and images for: Inhibition of U4 snRNA in Human Cells Causes the Stable Retention of Polyadenylated Pre-mRNA in the Nucleus
Source: PLoS One. 2014 May 5;9(5):e96174. doi: 10.1371/journal.pone.0096174 (PMC4010461; doi:10.1371/journal.pone.0096174)

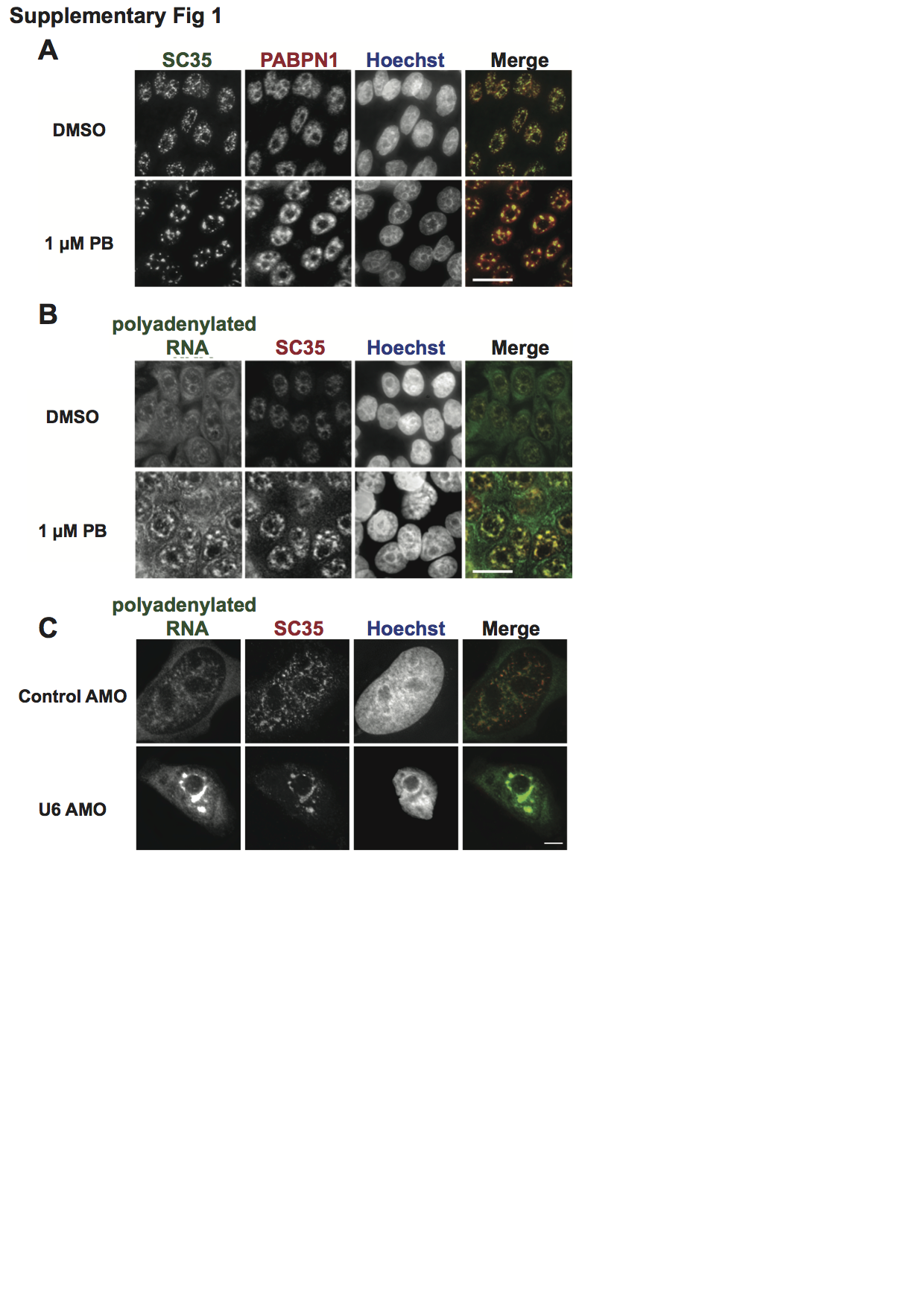

Supplement: Figure S1 — Pladienolide B or U6 AMO treatment results in the accumulation of polyadenylated RNA within enlarged nuclear speckles. A. IF of SC35 and PABPN1 in cells treated with DMSO or the splicing inhibitor Pladienolide B (PB). Scale bar is 20 µm. B. FISH and IF of polyadenylated RNA and SC35 respectively in cells treated with DMSO or the splicing inhibitor PB. Scale bar is 20 µm. C. FISH and IF of polyadenylated RNA and SC35 respectively in cells treated with control or U6 AMO (10 nmol). Scale bar is 5 µm. (TIFF) [file pone.0096174.s001.tiff]

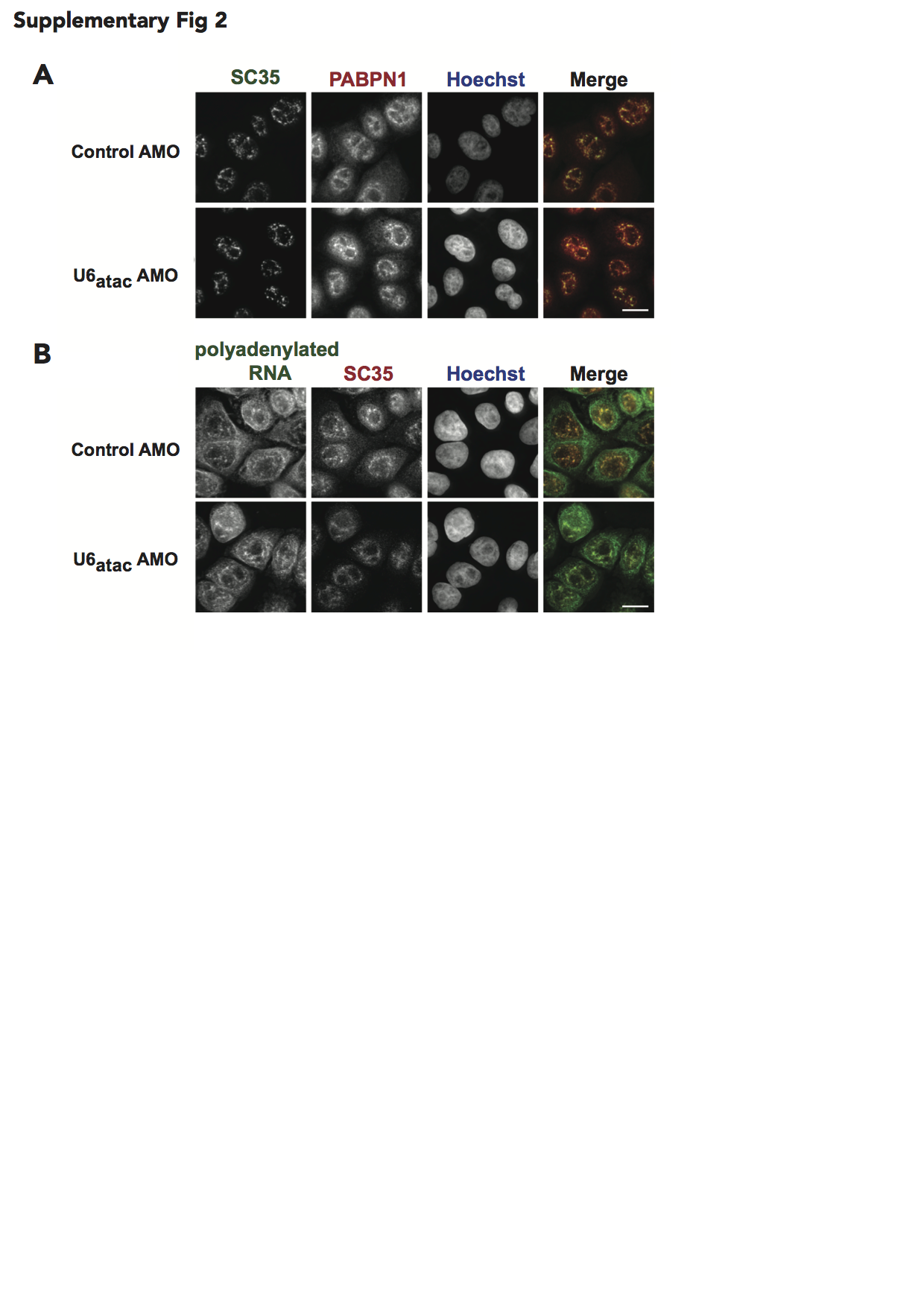

Supplement: Figure S2 — U6atac inhibition does not result in enlarged nuclear speckles. A. IF of SC35 and PABPN1 in cells treated with control or U6atac AMO (10 nmol). Scale bar is 15 µm. B. FISH and IF of polyadenylated RNA and SC35 respectively in cells treated with control or U6atac AMO (10 nmol). Scale bar is 15 µm. (TIFF) [file pone.0096174.s002.tiff]

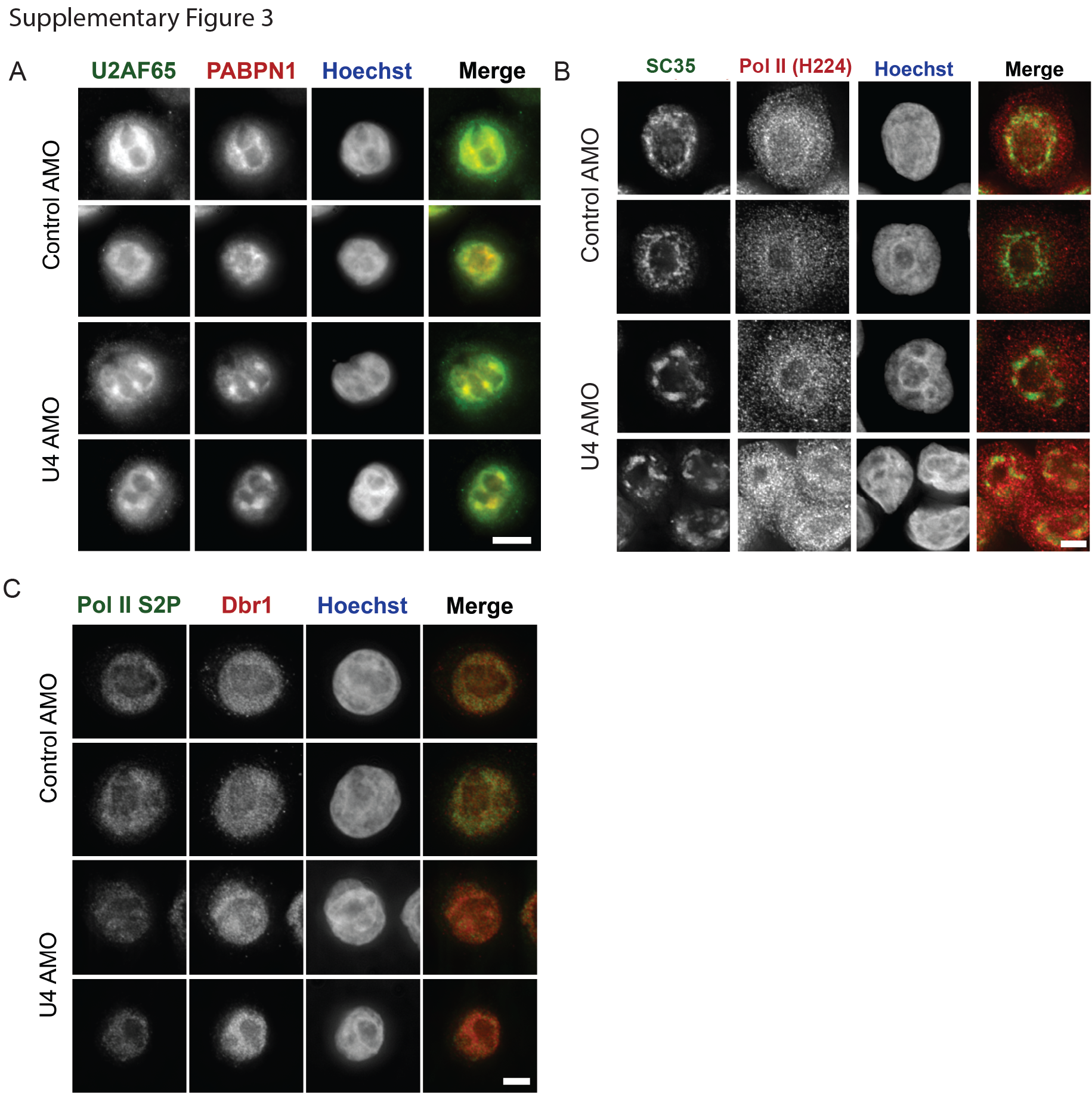

Supplement: Figure S3 — Alternative cell pictures accompanying main text figure 3 . A. IF of U2AF65 and PABPN1 in cells treated with control of U4 AMO (10 nmol). Two panels are shown per condition. Scale bar is 20 µm. B. IF of SC35 and total Pol II (H224) in cells treated with control of U4 AMO (10 nmol). Two panels are shown per condition. Scale bar is 5 µm. C. IF of Pol II S2P and Dbr1 in cells treated with control of U4 AMO (10 nmol). Two panels are shown per condition. Scale bar is 5 µm. (TIF) [file pone.0096174.s003.tif]

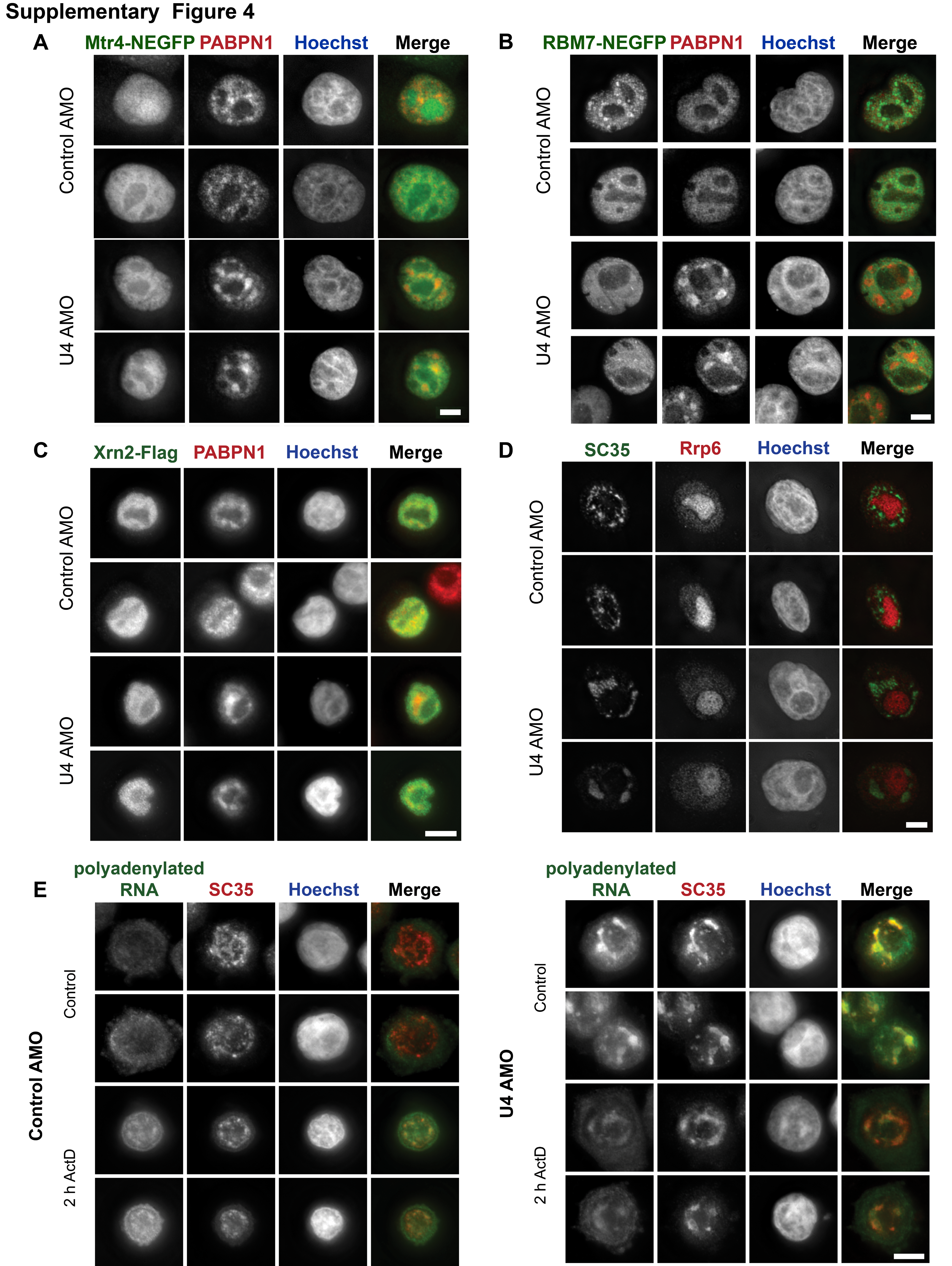

Supplement: Figure S4 — Alternative cell pictures accompanying main text figure 5 . A. IF of GFP-tagged Mtr4 and PABPN1 in cells treated with control of U4 AMO (10 nmol). Two panels are shown per condition. Scale bar is 5 µm. B. IF of GFP-tagged Rbm7 and PABPN1 in cells treated with control of U4 AMO (10 nmol). Two panels are shown per condition. Scale bar is 5 µm. C. IF of flag-Xrn2 and PABPN1 in cells treated with control of U4 AMO (10 nmol). Two panels are shown per condition. Scale bar is 15 µm. D. IF of SC35 and Rrp6 in cells treated with control of U4 AMO (10 nmol). Two panels are shown per condition. Scale bar is 5 µm. E. Poly(A)+ RNA FISH and SC35 IF in control (left-hand panels) and U4 AMO treated (right-hand panels) cells treated with ethanol (control) or Act D for two hours in cells treated with control of U4 AMO (10 nmol). Two panels are shown per condition. Scale bar is 15 µm. (TIF) [file pone.0096174.s004.tif]

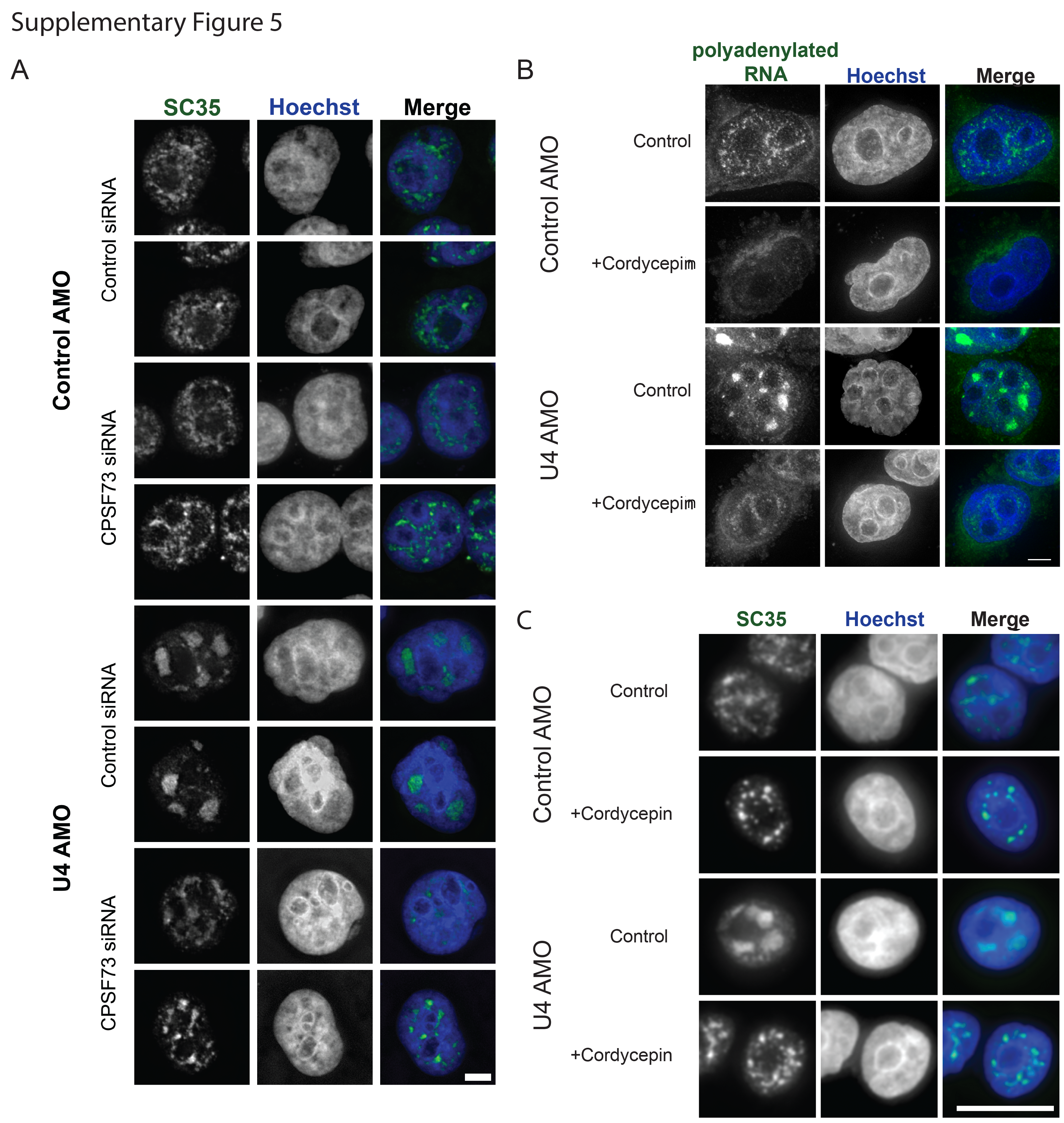

Supplement: Figure S5 — pre-mRNA cleavage and polyadenylation are required for the formation of enlarged speckles following splicing inhibition. A. Alternative pictures accompanying main text figure 6C: IF of SC35 in cells treated with control or CPSF73 siRNAs and, subsequently, with control or U4 AMO. Two data panels are shown. Scale bar is 5 µm. B. Poly(A)+ RNA FISH in control and U4 AMO treated cells treated with DMSO or the polyadenylation inhibitor cordycepin (CDY). CDY prevents the formation of enlarged poly(A)+ speckles following U4 AMO treatment. Scale bar is 5 µm. C. SC35 IF in control and U4 AMO treated cells treated with DMSO or the polyadenylation inhibitor cordycepin (CDY). CDY prevents the formation of enlarged SC35-containing speckles following U4 AMO treatment. Scale bar is 20 µm. (TIF) [file pone.0096174.s005.tif]
